# Population Attributable Risk Fractions of Maternal Overweight and Obesity for Adverse Perinatal Outcomes

**DOI:** 10.1038/srep22895

**Published:** 2016-03-10

**Authors:** Natasha MacInnis, Christy G. Woolcott, Sarah McDonald, Stefan Kuhle

**Affiliations:** 1 Perinatal Epidemiology Research Unit, Depts. of Obstetrics & Gynaecology and Pediatrics, Dalhousie University, Halifax, NS, Canada; 2 Department of Obstetrics & Gynecology, Division of Maternal-Fetal Medicine, McMaster University, Hamilton, ON, Canada; 3 Department of Radiology, McMaster University, Hamilton, ON, Canada; 4 Department of Clinical Epidemiology and Biostatistics, McMaster University, Hamilton, ON, Canada

## Abstract

The objective of the current study was to determine the proportion of adverse perinatal outcomes that could be potentially prevented if maternal obesity were to be reduced or eliminated (population attributable risk fractions, PARF); and the number needed to treat (NNT) of overweight or obese women to prevent one case of adverse perinatal outcome. Data from the Atlee Perinatal Database on 66,689 singleton infants born in Nova Scotia, Canada, between 2004 and 2014, and their mothers were used. Multivariable-adjusted PARFs and NNTs of maternal pre-pregnancy weight status were determined for various perinatal outcomes under three scenarios: If all overweight and obese women were to i) become normal weight before pregnancy; ii) shift down one weight class; or iii) lose 10% of their body weight, significant relative reductions would be seen for gestational diabetes mellitus (GDM, 57/33/15%), hypertensive disorders of pregnancy (HDP, 26/16/6%), caesarean section (CS, 18/10/3%), and large for gestational age births (LGA, 24/14/3%). The NNT were lowest for the outcomes GDM, induction of labour, CS, and LGA, where they ranged from 13 to 73. The study suggests that a substantial proportion of adverse perinatal outcomes may be preventable through reductions in maternal pre-pregnancy weight.

Around one in five women of childbearing age in Canada are obese with Nova Scotia having the highest prevalence among the provinces at 26%^1^. Obese women are known to be at increased risk of pregnancy complications (e.g., gestational diabetes mellitus (GDM), hypertensive disorders of pregnancy (HDP), and caesarean delivery) as well as adverse neonatal outcomes (e.g., large for gestational age (LGA), respiratory depression, and fetal distress)^2^,^3^,^4^,^5^. While the associations of maternal obesity with short- and long-term health outcomes have been studied, few studies have quantified the population disease burden associated with maternal obesity in pregnancy^6^,^7^,^8^.

Two metrics could be useful when considering the perinatal effects of maternal obesity at a population level. Population attributable risk fractions (PARFs) provide estimates of the proportion of adverse outcomes that could be potentially prevented if a specific causal factor, such as obesity, were to be reduced or eliminated^9^. The calculation of PARFs considers both the strength of the relationship between maternal obesity and adverse outcomes, as well as the prevalence of maternal obesity in the population. The number needed to treat (NNT) provides an estimate of the average number of women in whom obesity would need to be modified (i.e., prevented or successfully treated) to avoid one adverse outcome^10^. Knowing PARFs and NNTs of a population would enable policy makers to estimate cost savings that may be realized with improvements in maternal health or to conduct cost-effectiveness analyses of public health intervention strategies. The objective of the current study was to determine PARFs and NNTs of excess pre-pregnancy weight for a range of adverse perinatal outcomes. We wished to determine PARFs under various weight change scenarios, ranging from the most extreme (in which all women with excess weight shift to a normal weight) to a more pragmatic scenario (in which all women with excess weight lose 10% of their body weight).

## Methods

### Study design and population

The present study was a population-based retrospective cohort study of women without pre-existing diabetes or hypertension in Nova Scotia who gave birth to a singleton infant between 23 and 44 weeks gestational age during the study period from January 1, 2004 to December 31, 2014. Information on the mothers and their infants was obtained from the Nova Scotia Atlee Perinatal Database (NSAPD). The NSAPD has collected information on all births to mothers who were resident in the Canadian province of Nova Scotia since 1988. Information collected includes socio-demographic variables, medical conditions, reproductive history, delivery events, and neonatal outcomes. Trained coders enter these data from standard clinical forms including a Prenatal Record to document prenatal care and antenatal factors and forms completed at hospital delivery admission, birth, and discharge. The Reproductive Care Program of Nova Scotia administers the NSAPD, maintains the coding system, and ensures the quality, integrity and security of the data. Periodic abstraction and validation studies form an ongoing data quality assurance program and have shown that the data are accurate and reliable^11^. The study was approved by the IWK Health Centre Research Ethics Board (file # 1015714), Halifax, NS, Canada, and the Data Access Committee of the Reproductive Care Program of Nova Scotia. All procedures performed were in accordance with the ethical standards of the institutional research committee and with the *Tri-Council Policy Statement: Ethical Conduct for Research Involving Humans, December 2014*.

### Main exposure

The main exposure of interest was pre-pregnancy weight status based on body mass index (BMI), calculated from height and weight information collected by self-report or measured at the first prenatal visit. Maternal BMI was categorized as underweight (<18.5 kg/m^2^), normal weight (18.5 to <25 kg/m^2^), overweight (25 to <30 kg/m^2^), obesity class I (30 to <35 kg/m^2^), obesity class II (35 to <40 kg/m^2^), or obesity class III (≥40 kg/m^2^).

### Outcomes

We examined both maternal and neonatal adverse outcomes. Maternal outcomes included GDM, HDP (including gestational hypertension and pre-eclampsia), induction of labour, and caesarean section. Neonatal outcomes comprised LGA (weight above the 90^th^ percentile for gestational age and sex^12^), small for gestational age (SGA; weight below the 10^th^ percentile for gestational age and sex^12^), 5-minute Apgar score ≤7, venous cord blood pH ≤ 7.10, respiratory distress syndrome (RDS, excluding transient tachypnea of the newborn), admission to the neonatal intensive care unit (NICU), and fetal or neonatal (within the first 28 days of life) death.

### Confounders

We used demographic and maternal characteristics recorded in the NSAPD as potential confounders. These included maternal age at delivery, parity, area of residence (urban or rural, based on the Canadian postal code), and area-level income quintile derived from Census of Canada information^13^.

### Statistical Analysis

Sample characteristics were summarized by weight status categories. Relative risks (RR) for the associations between maternal excess weight and the perinatal outcomes of interest were derived from odds ratios estimated from logistic regression models (for perinatal outcomes with a prevalence <10%) and from prevalence ratios estimated from Poisson regression models with robust standard errors (for perinatal outcomes with a prevalence ≥10%)^14^. All regression models were adjusted for maternal age, parity, area of residence, and area-level income quintile.

The proportion of cases that could be prevented (i.e., PARFs) were estimated for three scenarios of population-level changes in maternal pre-pregnancy weight status: 1) Overweight and obese women become normal weight; 2) Overweight and obese women move down one weight status category (i.e., overweight women become normal weight, obese class I women become overweight, and so forth); and 3) Overweight and obese women lose 10% of their body weight. Within each of these scenarios, the PARF for each of the outcomes was calculated using the standard formula^15^:


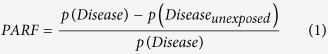


where *p(Disease)* is the observed prevalence (probability) of the outcome in the population and *p(Disease*_*unexposed*_) is the predicted prevalence of the outcome after a shift to the new distribution of maternal weight status has been achieved. Confidence intervals for the PARFs were calculated using a bootstrapping procedure with 1000 replications.

The NNT, the number of women with excess weight who would need to move to the next lower weight status category to prevent one case of each perinatal outcome of interest, was calculated as the inverse of the absolute risk difference between adjacent weight status categories from the multivariable-adjusted regression models^10^.

Stata/SE 13 (Stata Corp., College Station, TX, US) with the user-written packages *punaf* (PARF)^16^ and *adjrr* (NNT)^17^ was used to perform the statistical analysis.

## Results

A total of 91,459 singleton births between 23 and 44 weeks gestational age were recorded in the NSAPD between 2004 and 2014. Information on weight and height was available for 68,000 women. After excluding 1311 women with pre-existing diabetes or hypertension, the final sample size was 66,689 women. Twenty-two percent of women in our sample were obese, 24% were overweight, 50% were normal weight, and 4% were underweight. Compared to normal weight women, obese women were more likely to have higher parity, live in rural areas, be of lower socioeconomic status, have excessive gestational weight gain^18^, and have larger babies. The sociodemographic and clinical characteristics of the women by pre-pregnancy weight status are shown in Table 1.

The adjusted RRs shown in Table 2 suggest that overweight and obese women were at higher risk for all outcomes but the delivery of an SGA infant compared to normal weight women. The RRs were highest for GDM, HDP, LGA birth, and caesarean section. With very few exceptions, risk increased with increasing weight status.

**Figure 1:**
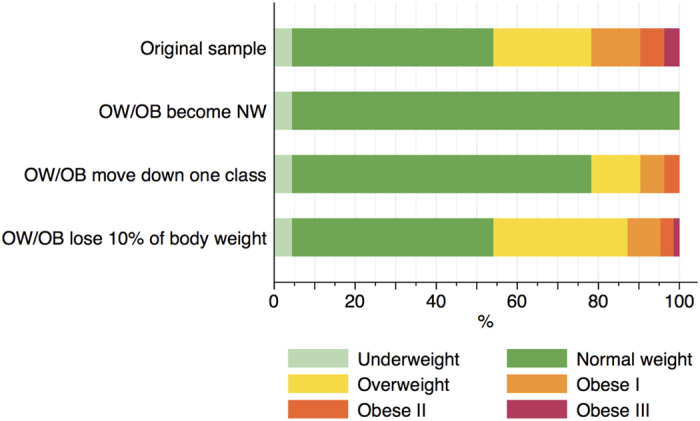
Distribution of maternal weight status in the original sample and under the three hypothetical scenarios. Abbreviations: *NW* Normal weight; *OB* Obese; *OW* Overweight.

The distribution of maternal weight status in the original sample and under the three hypothetical scenarios is shown in Fig. 1. The PARFs in Table 3 showed that more than half (57%) of GDM cases, 26% of HDP cases, 18% of caesarean sections, and 24% of LGA births may potentially be prevented if all overweight and obese women became normal weight, while the number of SGA births would increase by 13% under that scenario (from 7.5 to 8.4%). Assuming that all overweight and obese women lose 10% of their body weight, the greatest reductions would be expected for GDM (15%), HDP (6%), LGA infants (3%), and caesarean section (3%).

The numbers of women who would need to move down one weight status category to prevent one adverse outcome (i.e. NNTs) were lowest for the outcomes GDM, induction of labour, caesarean section, and LGA infants, where they ranged from 13 to 73 (Table 4). For HDP, cord pH ≤ 7.10, and RDS the potential benefits were greatest (i.e., NNT were lowest) for women in obesity class III moving to class II, whereas for the remainder of the outcomes, no clear pattern in the magnitude of the NNT was observed across the weight status categories.

## Discussion

Using a population-based cohort of women and their offspring from the Canadian province of Nova Scotia, we examined different weight loss scenarios to determine what proportion of adverse perinatal outcomes could potentially be prevented if overweight and obesity in women of childbearing age were reduced or eliminated. We found that over 20% of GDM, HDP, and LGA births potentially could be prevented with the elimination of overweight and obesity in this population. The number of women that would need to reduce their weight status by one category to prevent one case of adverse perinatal outcomes was lowest for GDM, induction of labour, caesarean section, and LGA infants, where they ranged from 13 to 73.

The risks for adverse perinatal outcomes associated with obesity are well described. Results from the current study were similar to another Canadian study, the All Our Babies Cohort, a prospective pregnancy cohort (n = 1996) in Calgary, Alberta^19^,^20^. Although the prevalence of obesity in the current study (22%) was considerably higher than in the convenience sample of Calgary women (11%), the proportion of overweight women was comparable between the two studies (approx. 25%). The risk of GDM, HDP, induction of labour, and caesarean section in obese vs. normal weight women was similar to that found in the present study^19^, as were the risk estimates for the neonatal outcomes SGA, LGA, NICU admission, and low Apgar score^20^. A study in a population-based sample of 6421 women from the Canadian Maternity Experiences Survey 2005/2006 (21% overweight, 13% obese) also reported similar risk estimates for caesarean section, LGA, and SGA in obese compared to normal weight women. These trends in the risk for maternal and neonatal outcomes in obese women can also be found in other settings and countries^21^ and highlight the considerable public health and clinical significance of maternal obesity.

Odds ratios offer an estimate of the strength of an association but they cannot provide information on the population disease burden that is due to the underlying exposure. This information can be obtained by determining the PARFs, which consider both the strength of an association and the prevalence of the exposure in the population. While PARFs are commonly used to compare the preventive potential of different risk factors for an outcome, the PARFs of maternal overweight and obesity in the current paper can help our understanding of the extent to which various adverse perinatal outcomes can be reduced if maternal excess weight were reduced or eliminated. This information can help policy makers to weigh prevention and intervention costs against health and economic benefits. So far, only few studies have determined PARF’s for maternal excess weight in pregnancy^6^,^7^,^22^,^23^,^24^,^25^ and the current study is the largest and most comprehensive study to date. A Canadian study in a population-based sample of 5591 women showed that 10.1% of caesarean section deliveries and 15.4% of LGA births could be attributed to overweight and obesity^6^,^7^ but the study was limited by the self-report of maternal pre-pregnancy BMI six months after pregnancy. The lower PARFs compared to the current study may be due to the lower prevalence of overweight and obesity in that study, and the potential for exposure misclassification as women self-reported their pre-pregnancy weight status about 6 months after delivery. A recent study from the UK using a sample of 23,668 singleton deliveries from two London hospitals found that 29% of GDM, 12% of caesarean section deliveries, 7% of macrosomia, and 6% of NICU admissions could be attributed to being obese^26^. Of note, the UK study found considerable differences in PARFs between ethnic groups with black women having consistently higher PARFs than other ethnic groups^26^.

In addition to the “traditional” PARF calculations, which are based on the complete elimination of an exposure, the current study also determined the potential reduction of adverse perinatal outcomes that may be achieved under scenarios with less dramatic changes, such as moving down one weight status category or losing 10% of body weight. The scenarios presented provide policy makers with more realistic estimates when weighing health and economic benefits against the cost of obesity prevention strategies. It should also be noted that the PARF estimates behave linearly. For example, we estimated herein that if all overweight and obese women lost 10% of their body weight, the expected reduction in the prevalence of GDM would be 14.5%; therefore, if only 20% of overweight and obese women lost 10% of their body weight, the expected reduction in GDM would be approximately 2.9% (0.2 × 14.5%).

Although the magnitude of these reductions for individual outcomes may appear small, it is important to consider that the health and economic benefits may be additive across outcomes. Maternal overweight and obesity may also be associated with additional outcomes and the requirement of more intensive care and specialized resources that are difficult to capture in registry-based cohort studies. For example, maternal obesity is strongly associated obesity in the offspring^27^, which in turn is associated with a disease and economic burden of its own^28^,^29^. As another example, maternal overweight and obesity may also cause additional issues that are difficult to capture in registry-based cohort studies. For example, overweight and obese women are more likely to experience difficulties with obstetrical anaesthesia due to their decreased pulmonary and cardiovascular reserve, as well as the increased technical difficulty for the anaesthesiologist when placing the epidural^30^, which may be associated with a substantial additional burden on health care resources.

Another way to examine how effective an intervention may be in reducing adverse outcomes is to examine the NNT to eliminate one case of adverse outcome. These calculations allow for interventions to be targeted by weight class status in order to achieve maximum adverse outcome reduction. Our findings show that the NNT varies with weight class and outcome without a consistent pattern. For example, fewer women with class I or class II obesity than overweight women would need to move down one weight status category to prevent one case of GDM, whereas to prevent one case of HDP far fewer women with obesity Class III would need to move to class II obesity than a reduction in weight status among other overweight or obese women. Approximately 20 women in any of the weight status categories would need to move to a lower weight status to prevent the need for one induction of labour or caesarean section.

The strengths of the current study include the use of a large population-based cohort that allowed for a stratified analysis by obesity class, and the broad range of high quality perinatal data entered by trained staff. Since unadjusted PARFs or NNTs may be falsely high or low if confounding is present, the ability to adjust for relevant confounders and calculate multivariable-adjusted PARFs and NNTs is another strength of the current analysis. Notwithstanding the strengths of the current study, several limitations exist and need to be acknowledged. The PARFs and NNTs are calculated on the assumption of a causal link between the exposure and outcome. This assumption may not hold true in the presence of residual or unmeasured confounders. However, substantial evidence exists to support the link between the exposure and outcomes examined in the current study and confounders were adjusted for accordingly. In addition, maternal height and weight were self-reported data, which could possibly lead to misclassification of weight status, but maternal self-report of pre-pregnancy weight has been shown to agree closely with measured weight^31^,^32^. We were also limited by missing data for maternal BMI. Maternal height is not reported by a small proportion of health care providers in the province but it is unclear if the associations of interest differ between women with and without missing BMI. Lastly, since PARFs are dependent on the prevalence of the exposure, the findings from the current study may not apply to settings with a lower prevalence of obesity.

## Conclusions

This study has for the first time examined hypothetical scenarios in an attempt to provide a realistic perspective of the positive benefits of weight loss in women of childbearing age and used the NNT concept to estimate the number of women in each weight status category who would need to successfully lose weight to avoid one case of the adverse perinatal outcome under study. The largest reductions in adverse perinatal outcomes through reduction of maternal excess weight may be achieved for GDM and HDP, caesarean section, and LGA births. The NNT for maternal weight loss were lowest for GDM, induction of labour, caesarean section, and LGA birth, where they ranged from 13 to 73. Results from these analyses will enable policy makers to weigh health and economic benefits against the cost of obesity prevention strategies and allow for interventions to be targeted by weight class status in order to achieve maximum adverse outcome reduction.

## Additional Information

**How to cite this article**: MacInnis, N. *et al.* Population Attributable Risk Fractions of Maternal Overweight and Obesity for Adverse Perinatal Outcomes. *Sci. Rep.* **6**, 22895; doi: 10.1038/srep22895 (2016).

## Figures and Tables

**Table 1:** Sociodemographic and clinical characteristics of Nova Scotian women with a singleton birth between 2004 and 2014, stratified by pre-pregnancy weight status (n = 66,689).

	Underweight	Normal weight	Overweight	Obese I	Obese II	Obese III
	4% (2981)	50% (33,127)	24% (16,101)	12% (8089)	6% (3942)	4% (2449)
Maternal age [years]	25.7 (5.8)	28.5 (5.7)	28.9 (5.5)	28.9 (5.4)	28.9 (5.2)	29.1 (5.1)
Maternal weight [kg]	47.7 (4.6)	59.5 (6.5)	73.5 (7.2)	87.1 (8.3)	100.6 (9.2)	118.9 (13.5)
Parity						
0	43%	40%	35%	33%	32%	32%
1	30%	32%	33%	32%	33%	33%
2	15%	16%	18%	18%	19%	19%
3+	13%	12%	15%	16%	17%	17%
Smoking on admission	32%	16%	16%	17%	17%	16%
Rural residence	26%	26%	29%	31%	34%	35%
Area-level household income quintile						
Quintile 1	24%	18%	19%	21%	22%	24%
Quintile 2	21%	20%	21%	22%	25%	24%
Quintile 3	21%	22%	23%	25%	24%	23%
Quintile 4	21%	23%	22%	20%	19%	19%
Quintile 5	13%	17%	14%	12%	10%	10%
Gestational weight gain*						
Adequate	37%	32%	18%	16%	19%	21%
Inadequate	21%	17%	9%	13%	21%	32%
Excessive	42%	51%	73%	71%	60%	46%
Gestational age [weeks]	38.8 (1.9)	38.9 (1.8)	39.0 (1.7)	39.0 (1.8)	39.0 (1.8)	39.0 (1.7)
Birth weight [g]	3216 (535)	3407 (524)	3505 (536)	3553 (568)	3588 (561)	3614 (573)

^*^Gestational weight gain was categorized as being above, within or below the IOM recommendations of 12.5 to 18 kg for underweight, 11.5 to 16 kg for normal weight, 7 to 11.5 kg for overweight, and 5 to 9 kg for obese women based on their prepregnancy BMI^18^.

**Table 2:** Prevalence of adverse maternal and neonatal outcomes by pre-pregnancy weight status, and relative risks (RR)* with 95% confidence intervals (CI) for the association between pre-pregnancy weight status and adverse maternal and neonatal outcomes in Nova Scotian women with a singleton birth between 2004 and 2014 (n = 66,689).

	Underweight		Normal weight		Overweight		Obese I		Obese II		Obese III	
	%	RR (95% CI)	%	RR (95% CI)	%	RR (95% CI)	%	RR (95% CI)	%	RR (95% CI)	%	RR (95% CI)
Maternal												
Gestational diabetes mellitus	1.6	**1.12** (0.81;1.56)	1.9	**1.00** (ref)	4.2	**2.36** (2.04;2.61)	8.4	**4.82** (4.23;5.49)	12.2	**7.17** (6.20;8.30)	15.0	**8.96** (7.65;10.49)
Hypertensive disorders of pregnancy	1.0	**0.92** (0.62;1.37)	1.2	**1.00** (ref)	1.7	**1.60** (1.35;1.89)	2.3	**2.20** (1.81;2.66)	2.3	**2.22** (1.73;2.86)	4.0	**3.90** (3.04;5.00)
Induction	22.8	**0.95** (0.88;1.03)	24.7	**1.00** (ref)	29.6	**1.24** (1.20;1.28)	34.0	**1.44** (1.38;1.49)	37.5	**1.61** (1.53;1.69)	39.9	**1.69** (1.60;1.79)
Caesarean section	16.0	**0.80** (0.73;0.89)	21.8	**1.00** (ref)	28.0	**1.29** (1.24;1.34)	32.8	**1.51** (1.44;1.58)	36.9	**1.69** (1.60;1.79)	45.1	**2.05** (1.94;2.19)
Neonatal												
Large for gestational age	5.8	**0.58** (0.49;0.68)	10.9	**1.00** (ref)	16.0	**1.45** (1.38;1.53)	19.6	**1.78** (1.68;1.90)	21.4	**1.91** (1.76;2.07)	24.8	**2.29** (2.10;2.49)
Small for gestational age	15.1	**1.70** (1.52;1.89)	8.1	**1.00** (ref)	6.3	**0.81** (0.75;0.88)	6.1	**0.77** (0.69;0.85)	5.3	**0.69** (0.59;0.80)	5.3	**0.66** (0.55;0.80)
5 min Apgar ≤7	3.2	**1.22** (0.96;1.54)	2.8	**1.00** (ref)	3.0	**1.11** (0.98;1.26)	3.4	**1.28** (1.10;1.49)	3.2	**1.20** (0.97;1.48)	4.6	**1.86** (1.50;2.31)
Neonatal intensive care unit admission	11.9	**1.28** (1.11;1.47)	9.7	**1.00** (ref)	10.5	**1.10** (1.02;1.18)	11.1	**1.20** (1.09;1.32)	12.7	**1.40** (1.24;1.57)	13.6	**1.53** (1.32;1.77)
Cord pH ≤ 7.10	1.9	**1.04** (0.75;1.44)	1.9	**1.00** (ref)	2.3	**1.20** (1.03;1.40)	2.7	**1.45** (1.20;1.74)	2.5	**1.32** (1.02;1.72)	3.3	**1.92** (1.47;2.51)
Respiratory distress syndrome	4.4	**1.16** (0.95;1.42)	3.9	**1.00** (ref)	4.5	**1.16** (1.04;1.28)	5.0	**1.33** (1.17;1.52)	5.1	**1.36** (1.14;1.61)	6.0	**1.70** (1.40;2.06)
Fetal/neonatal death	0.4	**1.45** (0.80;2.63)	0.3	**1.00** (ref)	0.3	**0.98** (0.68;1.41)	0.4	**1.19** (0.78;1.81)	0.4	**1.46** (0.86; 2.49)	0.5	**1.16** (0.56;2.40)

^*^adjusted for maternal age, area-level income quintile, area of residence, and parity.

**Table 3:** Population attributable risk fractions - proportion of adverse perinatal outcomes potentially preventable under three hypothetical weight loss scenarios in Nova Scotian women with a singleton birth between 2004 and 2014 (n = 66,689).

	All overweight and obese women become normal weight	All overweight and obese women move down one weight status category	All overweight and obese women lose 10% of their body weight
	PARF [%] (95% CI)	PARF [%] (95% CI)	PARF [%] (95% CI)
Maternal			
Gestational diabetes mellitus	57.1 (54.2, 59.9)	32.6 (30.9, 34.3)	14.5 (13.2, 15.7)
Hypertensive disorders of pregnancy	26.1 (20.6, 31.6)	15.8 (12.7, 18.9)	5.6 (3.7, 7.4)
Induction	12.9 (11.7, 14.0)	7.1 (6.5, 7.8)	2.2 (1.9, 2.5)
Caesarean section	17.7 (16.4, 18.9)	10.1 (9.4, 10.8)	3.1 (2.8, 3.5)
Neonatal			
Large for gestational age	24.3 (22.5, 26.0)	13.5 (12.5, 14.4)	3.4 (2.9, 4.0)
Small for gestational age	−12.6 (−14.9, −10.2)	−6.7 (−8.0, −5.4)	−0.8 (−1.4, −0.2)
Neonatal intensive care unit admission	6.7 (4.7, 8.8)	3.9 (2.7, 5.0)	1.5 (0.84, 2.1)
Cord pH ≤ 7.10	12.0 (6.7, 17.3)	7.1 (4.4, 9.8)	2.3 (0.6, 4.0)
Respiratory distress syndrome	10.0 (6.6, 13.3)	5.8 (4.0, 7.6)	1.7 (0.7, 2.8)
Fetal/neonatal death	5.3 (−6.9, 17.4)	3.5 (−3.0, 10.0)	2.5 (−0.8, 5.9)

**Table 4:** Number needed to treat (NNT) by pre-pregnancy weight status category to prevent one case of adverse maternal or perinatal outcomes based on data from Nova Scotian women with a singleton birth between 2004 and 2014 (n = 66,689).

	OW to NW NNT	OB I to OW NNT	OB II to OB I NNT	OB III to OB II NNT
Maternal				
Gestational diabetes mellitus	42	24	28	40
Hypertensive disorders of pregnancy	146	149	minimal effect^1^	55
Induction	17	21	24	51
Caesarean section	16	21	25	13
Neonatal				
Large for gestational age	20	28	73	24
Small for gestational age	−66†	−292†	−151†	−529†
5 min Apgar ≤7	333	229	−459	59
Neonatal intensive care unit admission	132	124	71	104
Cord pH ≤ 7.10	261	219	−433	91
Respiratory distress syndrome	177	156	minimal effect^1^	83
Fetal/neonatal death	minimal effect^1^	minimal effect^1^	minimal effect^1^	−983

*Adjusted for maternal age, area-level income quintile, area of residence, and parity.

^†^Negative values represent the number needed to harm.

^1^Indicates absolute NNT values ≥1000 (corresponding to an absolute risk difference of 0.1%).
